# Species-specific multiplex PCR for the diagnosis of *Brucella ovis*, *Actinobacillus seminis*, and *Histophilus somni* infection in rams

**DOI:** 10.1186/1746-6148-9-51

**Published:** 2013-03-21

**Authors:** Valéria S Moustacas, Teane MA Silva, Luciana F Costa, Mariana N Xavier, Custódio A Carvalho, Érica A Costa, Tatiane A Paixão, Renato L Santos

**Affiliations:** 1 Departamento de Clínica e Cirurgia Veterinárias, Escola de Veterinária, Universidade Federal de Minas Gerais, Av. Antônio Carlos, 6627, 31270-901, Belo Horizonte, MG Brazil; 2 Departamento de Patologia Geral, Instituto de Ciências Biológicas da Universidade Federal de Minas Gerais, Av. Antônio Carlos, 6627, Belo Horizonte, MG 31270-901, Brazil

**Keywords:** *Actinobacillus seminis*, *Brucella ovis*, *Histophilus somni*, Epididymitis, Multiplex polymerase chain reaction

## Abstract

**Background:**

Infectious ovine epididymitis results in substantial economic losses worldwide due to reproductive failure and culling of breeders. The most common causative agents of these infections are *Brucella ovis*, *Actinobacillus seminis*, and *Histophilus somni.* The aim of this study was to develop a multiplex PCR assay for simultaneous detection of *Brucella ovis*, *Actinobacillus seminis*, and *Histophilus somni* with species-specific primers applied to biological samples for molecular diagnosis of these infections.

**Results:**

The multiplex assay was capable of detecting *B. ovis*, *A. seminis*, and *H. somni* DNA simultaneously from genomic bacterial DNA samples and pool of semen samples from experimentally infected rams. The method was highly specific since it did not amplify DNA from other bacterial species that can potentially cause epididymitis in rams as well as species phylogenetically related to *B. ovis.* All negative control samples were negative in PCR multiplex assay. Urine can be used as an alternative to semen samples.

**Conclusions:**

The species-specific multiplex PCR assay developed in this study can be successfully used for the detection of three of the most common bacterial causes of ovine epididymitis.

## Background

Infectious ovine epididymitis may be caused by a variety of microorganisms including *Actinobacillus lignieresi*, *Trueperella (Arcanobacterium) pyogenes*, *Chlamydia psittaci*, *Corynebacterium pseudotuberculosis*, *Escherichia coli*, *Mannheimia haemolytica*, *Pasteurella multocida*, and *Yesinia pesudotuberculosis*. However, the most common causative agents of these infections are *Brucella ovis*, *Actinobacillus seminis*, and *Histophilus somni*[1]. *A. seminis* is a component of the normal flora of the prepucial mucosa, but it can act as opportunistic pathogen causing ascending infection which may lead to epididymitis and orchitis, particularly in young rams [1,2]. *A. seminis* infections usually progresses asymptomatically during the early stages being diagnosed only when disease is established. *Histophilus somni*, previously described as *Haemophilus somnus*, *Haemophilus agni*, and *Histophilus ovis*, is naturally present in the mucosal surfaces of cattle, goats, and sheep [3]. Similarly to *A. seminis*, *H. somni* also may act as an opportunistic pathogen, but in addition to epididymitis, the infection may result in other clinical manifestations such as vaginitis, placentitis, pneumonia, meningoencephalitis, mastitis, synovitis, septicemia, and other reproductive disorders [4,5]. In rams, these bacteria can cause infection that resembles ovine brucellosis due to *B. ovis*[1,3] which is characterized by uni or bi-lateral epididymitis and orchitis that are associated with subfertility or infertility.

The diagnosis of *B. ovis* infection is based on clinical examination, serologic tests, and semen bacteriology [1,6]. Additionally, molecular methods based on amplification of *Brucella* spp. DNA have also been reported [7,8]. Importantly, more recently a species-specific PCR (polymerase chain reaction) has been developed for direct diagnosis *B. ovis* infections [9]. Serologic tests are not widely available for diagnosis of *A. seminis* and *H. somni* infections and, therefore, the diagnosis is commonly based on clinical evaluation and semen bacteriology, although PCR has been proposed as an alternative diagnostic method [10]. These infections are usually unresponsive to antibiotic treatment [11], resulting in considerable economic losses due to reproductive failure and culling of breeders [12].

Although a previous report described a multiplex PCR assay for detecting *B. ovis*, *A. seminis*, and *H. somni*[8], the primer combination employed in that study results in detection of *Brucella* spp. since it is a genus-specific primer pair. That may be a relevant limitation in areas where both *B. ovis* and *B. melitensis* are endemic in sheep, mostly due to the importance of *B. melitensis* for human public health, whereas *B. ovis* does not have zoonotic potential. Thus, a species-specific assay, as proposed in this study, would me more suitable under those conditions. Therefore, considering the importance of differential diagnosis, particularly for epidemiologic studies or eradication programs [13], the aim of this study was to develop and validate a multiplex species-specific PCR assay for simultaneous detection of *B. ovis*, *A. seminis*, and *H. somni*.

## Methods

### Experimental infections and sampling

Twenty crossed Santa Inês rams ranging 18 to 24 month-old, were used in this study. These rams were divided into two groups of 10 rams each. Rams were fed hay and commercial ration throughout the experiment, which took place in Belo Horizonte, Brazil (19.52°S, 43.57°W).

Both groups underwent a 2-month period of adaptation and training for semen sampling with artificial vagina. For semen sampling, estrus was induced in a crossbred ewe with 2 mg of estradiol cypionate (ECP – Pfizer, São Paulo, Brazil) intramuscularly 48 h before semen sampling. This protocol was repeated throughout the experiment whenever necessary. After the adaptation period, a first group of 10 rams were inoculated with 1 mL of a solution containing approximately 2.3 × 10^10^ CFU/mL (colony forming units) of *A. seminis* (strain ATCC 15768) injected into the left cauda epididymis [14].

The second group of 10 rams was inoculated with 1 mL of a solution containing approximately 1.0 × 10^9^ CFU/mL (colony forming units) of *H. somni* (strain 3384Y) injected into the left cauda epididymis [14]. These experimental infections were done consecutively and rams from different experimental groups never had contact with each other. Both experiments were approved by the Institutional Ethics Committee on Animal Experimentation (CETEA-UFMG, Protocol 285/2008 and 2/2010).

Semen, blood, urine, and preputial wash were obtained immediately before inoculation and every seven days post-infection (dpi), during 6 weeks, totaling seven time-points per group. Cross-contaminations among rams were prevented by using a plastic sterile and disposable liner inside the artificial vagina, connected to collection tube. Three rams of the first group had no libido during the experiment period and therefore they were subjected to electroejaculation for collecting semen samples [15]. Whole blood samples were obtained from the jugular vein by a vacuum collection system. At the same occasion, urine samples were obtained, and a prepucial wash was performed by introduction of 10 mL of a sterile PBS into the preputial cavity, followed by mucosal massage for 1 min and recovery of the suspension into a sterile 15 mL tube [16].

At six weeks post-infection, rams were euthanatized. In order to assess the suitability of various tissues for *A. seminis* and *H. somni* diagnosis, fragments of the tail, body, and head of both epididymis, testes, ampullas of the ductus deferens, seminal vesicles, bulbo-urethral glands, inguinal lymph nodes, medial iliac lymph nodes, prepuce, glans penis, spleen, liver, kidney, and urinary bladder were collected. Samples were placed in a 50 mL sterile tube containing 2 mL of sterile PBS solution for bacteriology and macerated with a homogenizer. Additional fragments of tissues were placed into cryotubes, snap frozen in liquid nitrogen, and stored at −80°C until DNA extraction.

For multiplex PCR evaluation, biological samples from *B. ovis* experimentally infected rams were obtained from a previous study [9,17].

### Negative control samples

As negative control, semen (n = 27), blood (n = 11), urine (n = 8), and prepucial wash (n = 8) samples from *B. ovis*-free healthy rams with no history of infertility were used.

### Bacteriology

For bacteriological isolation, 100 μL of each sample (tissue homogenates, semen, blood, urine, and preputial wash) were plated on GC medium (base medium for chocolate agar) (Becton Dickinson, Franklin Lakes, USA), supplemented with 1% bovine hemoglobin^c^, without antibiotics and incubated at 37°C for 48 h. For *H. somni* detection, 0.5% of yeast extract (Becton Dickinson) was added to the medium, and plates were cultured under an atmosphere with 5% CO_2_. Colonies were confirmed by specie-specific PCR for each agent [8-10].

### DNA extraction

DNA extraction was performed by the proteinase K and phenol/chlorophorm method as previously described [18] with 500 μL of fresh semen or blood samples, 1 mL of thawed urine or preputial wash samples, and approximately 800 μL of tissue homogenates. All DNA samples were stored at −20°C until amplification.

### Single PCR

Single PCR was performed using primer pairs previously described for detection of *A. seminis* (FWD 5′-CTTATCTTTCTTAAGCCCTGAC;-3′ and REV 5′-AAGAAAAAGACGAAGAGACATT-3′) and *H. somni* (FWD 5′-GAAGGCGATTAGTTTAAGAG-3′ and REV 5′-ACTCGAGCGTCAGTATCTTC-3′) [8,10]. PCR reactions were performed using 15 μL of a commercial PCR supermix, containing 22 mM Tris–HCl (pH 8.4), 55 mM KCl, 1.65 mM MgCl2, 220 μM dGTP, 220 μM dATP, 220 μM dTTP, 220 μM dCTP, 22 U recombinant Taq DNA Polymerase/mL (Invitrogen, São Paulo, Brazil) 1 μL of a 10 mM solution of each primer, and 1–3 μL of DNA template corresponding to 200–500 ng of DNA per reaction. Cycling parameters were the previously described for each target [8,10]. Reactions were carried out in a (Mastercycler, Eppendorf, Hamburg, Germany). Ultra-pure water was used replacing the DNA template as negative control. Genomic DNA extracted from *A. seminis* and *H. somni* pure cultures were used as positive controls. PCR products were analyzed by electrophoresis in 1% agarose gel (Invitrogen). Reactions were considered positive when they yielded products of 436 bp and 313 bp for primers targeting *A. seminis* or *H. somni*, respectively.

### Multiplex PCR assay

For multiplex assay, previously validated species-specific PCR primers for *B. ovis* detection (FWD 5′-GCCTACGCTGAAACTTGCTTTTG-3′ and REV 5′-ATCCCCCCATCACCATAACCGAAG-3′), which amplifies a 228 pb product were used [9]. *A. seminis* and *H. somni* primers were the same used for single PCR. A reaction solution containing 2 mM MgCl_2_ and 55°C as annealing temperature yielded products for all three targets without affecting the specificity of amplification.

Multiplex reactions were performed to a final volume of 31 μL, with 22 μL of PCR supermix (Invitrogen) containing 1.65 mM MgCl_2_, supplemented with 0.5 μL of 50 mM MgCl_2_, 1 μL of 25 mM of each primers, and 200–500 ng of DNA template. Cycling parameters were 94° for 2 min, followed by 35 cycles of 94°C for 30s, 55°C for 30s and 72°C for 1 min, and a final extension of 72°C for 6 min. PCR products were separated using electrophoresis in 1.8% agarose gel (Invitrogen). Amplified products were 218 bp, 436 bp, and 313 bp for *B. ovis*, *A. seminis*, and *H. somni*, respectively.

### Multiplex PCR sensitivity and specificity

Sensitivity of the multiplex PCR was assessed by performing reactions with various combinations of 0.2, 2, 20 or 200 ng of genomic DNA extracted from pure cultures of *B. ovis* (ATCC 25840), *A. seminis* (ATCC 15768) and *H. somni* (3384Y), resulting in 64 associations of different DNA concentrations of each agent.

In order to investigate possible influences of the biological specimen on the efficiency of DNA amplification, semen samples, which are the principal shedding route of these microorganisms, were spiked with ten-fold serial solutions of bacterial suspensions, ranging from 10^6^ to 10^0^ CFU/mL of each agent. In addition, to confirm that the assay was capable of detecting all three agents simultaneously in semen samples, three distinct positive samples from each experimental infection were pooled, subjected to DNA extraction, and multiplex PCR as described.

To assess the specificity of the multiplex PCR, genomic DNA from bacterial species that can potentially cause epididymitis in rams were used, including *B. ovis* (ATCC 25840), *A. seminis* (ATCC 15768), *H. somni* (3384Y), *Staphylococcus aureus* (ATCC 12600), *Manheimia haemolitica* (D0614057), *Corynebacterium pseudotuberculosis* (D0507204), and *Trueperella* (*Arcanobacterium*) *pyogenes* (D0602705) as well as an organism phylogenetically related to *B. ovis*, i.e. *Ochrobactrum anthropi* (ATCC 49188). PCR reactions were performed as described above.

### Statistical analysis

Frequency of positive samples by PCR and bacteriology were compared by Fisher’s exact test using GraphPad Instat software, version 3.10 and differences were considered significantly when P < 0.05. Agreement between diagnostic methods was evaluated by the Kappa test using GraphPad Quick Calcs software.

## Results

Samples from *B. ovis* experimentally infected rams were obtained from a previous study [9,17]. Results from *A. seminis* and *H. somni* experimental infections are described below.

### *Actinobacillus seminis* experimental infection

The experimental inoculation with *A. seminis* resulted in infection of all challenged rams since *A. seminis* was detected by single PCR or bacteriology in at least one time point during the course of experimental infection in all rams. None of the semen and blood samples were bacteriologically positive prior to inoculation (Time 0).

The frequency of *A. seminis* detection in semen and blood samples by PCR was significantly higher (P < 0.05) than the frequency of positivity by bacterial isolation. Conversely, no significant differences (P > 0.05) were observed between bacteriology and PCR when performed using urine or prepucial wash (Table 1). All samples used as negative control as negative to *A. seminis* in both techniques (i.e. PCR and bacteriology). In addition, agreement between techniques and kappa values were better for semen and urine (Table 1).

**Table 1:** **Frequency (%) of *Actinobacillus seminis *detection by PCR and bacteriology of semen, blood, urine, preputial wash samples from experimentally infected rams during six weeks of infection and from negative control**

**Sample**	**Infected rams**		**Negative control rams**		**Agreement (%)**^**c**^	**Kappa**^**c**^
	**Bacteriology**	**PCR**	**Bacteriology**	**PCR**		
Semen^d^	60.0% (42/60) ^a^	90.0% (55/60) ^b^	0.0% (0/27) ^a^	0.0% (0/27) ^a^	79.3	0.59
Blood^d^	0.0% (0/60) ^a^	77.1% (53/60) ^b^	0.0% (0/11) ^a^	0.0% (0/11) ^a^	25.4	0.00
Urine^d^	51.4% (36/60) ^a^	48.6% (34/60) ^a^	0.0% (0/8) ^a^	0.0% (0/8) ^a^	67.7	0.35
Preputial wash^d^	38.6% (27/60) ^a^	50.0% (33/60) ^a^	0.0% (0/8) ^a^	0.0% (0/8) ^a^	55.9	0.11

^a,b^ Different letters in the same row indicate statistically significant differences (P < 0.05) according to the Fisher’s exact test.

^c^ Kappa test values considering samples from experimental infection and negative controls.

^d^ These are cumulative results from samples collected weekly throughout the course of infection (up to 42 days post infection).

**Figure 1:**
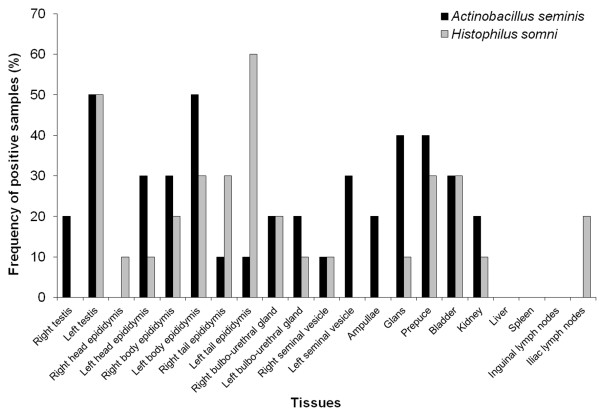
**Frequency (%) of *Actinobacillus seminis *and *Histophilus somni *detection by PCR in tissue samples from experimentally infected rams at 45 days post infection.**

Evaluation of tissue samples from experimentally infected rams demonstrated that 90% (9/10) of them had evidence of *A. seminis* infection either by PCR at 45 dpi (Figure 1). *A. seminis* was detected in 20.5% (43/210) of tissues samples by PCR. *A. seminis* was mostly detected by PCR in the left body of epididymis, left testis (50% each) (Figure 1). Notably, *A. seminis* was not detected in any liver, spleen, inguinal and iliac lymph nodes samples.

### *Histophilus somni* experimental infection

Intra-epididymal *H. somni* inoculation resulted in infection in 80% (8/10) of rams, since *H. somni* was detected by single PCR or bacteriology in at least one time point during the course of experimental infection in these rams, which were all bacteriologically and PCR negative prior to infection.

Bacteriology and PCR had 38.3% and 58.3% positivity in semen samples respectively (P < 0.05), with 82.9% of agreement between these techniques considered good. However, there was no *H. somni* detection by bacteriology in any of the 60 blood samples, whereas 10% of these samples were positive by PCR (P < 0.05) (Table 2). No significant differences (P > 0.05) were observed between bacteriology and PCR when performed using urine or prepucial wash samples. In addition, all samples used as negative control were negative to *H. somni* in PCR and bacteriology (Table 2).

**Table 2:** **Frequency (%) of *Histophilus somni *detection by PCR and bacteriology of semen, blood, urine, preputial wash and tissue samples from experimentally infected rams, during six weeks of infection and from negative control**

**Sample**	**Infected rams**		**Negative control rams**		**Agreement (%)**^**c**^	**Kappa**^**c**^
	**Bacteriology**	**PCR**	**Bacteriology**	**PCR**		
Semen^d^	38.3% (23/60) ^a^	58.3% (35/60) ^b^	0.0% (0/27) ^a^	0.0% (0/27) ^a^	86.2	0.70
Blood^d^	0.0% (0/60) ^a^	10.0% (6/60) ^a^	0.0% (0/11) ^a^	0.0% (0/11) ^a^	91.4	0.00
Urine^d^	40.0% (24/60) ^a^	23.3% (14/60) ^a^	0.0% (0/8) ^a^	0.0% (0/8) ^a^	73.5	0.36
Preputial wash^d^	35.0% (21/60) ^a^	30.0% (18/60) ^a^	0.0% (0/8) ^a^	0.0% (0/8) ^a^	72.1	0.32

^a,b^ Different letters in the same row indicate statistically significant differences (P < 0.05) according to the Fisher’s exact test.

^c^ Kappa test values considering samples from experimental infection and negative controls.

^d^ These are cumulative results from samples collected weekly throughout the course of infection (up to 42 days post infection).

At 45 dpi, PCR detected *H. somni* in various organs, particularly in the reproductive tract (Figure 1b). PCR detected *H. somni* DNA from 50% (10/20) of the left testis and 60% (6/10) of the left tail of the epididymis. Considering all tissues, PCR was positive in 29.5% (62/210) of the samples.

### Multiplex PCR sensitivity and specificity

**Figure 2:**
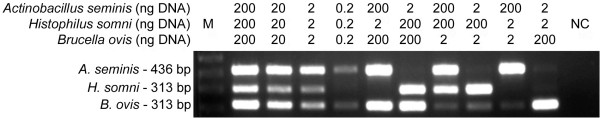
**Representative agarose gel electrophoresis resolving products from a multiplex PCR assay for detection of *Brucella ovis, Actinobacillus seminis*,** **and *Histophilus somni *with genomic DNA extracted from pure cultures.** M: molecular weight marker; NC: negative control without genomic DNA.

Analytical sensitivity of the multiplex PCR was assessed by using DNA templates containing only genomic bacterial DNA from the three agents, at various concentrations resulting in several combinations of concentration of genomic DNA from the three organisms (Figure 2). Considering the 64 different DNA combinations (Additional file 1: Table S1) from these three agents, the multiplex PCR proved to simultaneously detect all three agents when DNA concentrations are added to the reaction within the recommended standard amounts of template DNA. With concentrations of genomic DNA equal to or higher than 2 ng of DNA/reaction of each agent all organisms were detected (Figure 2). However, in some cases there was an impairment of the sensitivity. Inhibition of PCR also occurred when DNA concentration of one of the agents was a hundred fold higher that the other organisms. Therefore, when *A. seminis* and *B. ovis* or *H. somni* and *B. ovis* were at concentration of 200 ng of genomic DNA per reaction, and the third agent at 2 ng per reaction, there was inhibition of amplification of this third agent. However, even when the concentrations of *A. seminis* and *H. somni* were a hundredfold higher, amplification of *B. ovis* DNA was successful.

In addition, DNA was extracted from semen samples free of the three agents, and then spiked with DNA from each one of the three microorganisms separately (Table 3). Compared to single PCR, the specific multiplex PCR assay had the same detection limit for *B. ovis* (10^4^ CFU/mL) in spiked semen samples. The detection limit of *A. seminis* decreased 10 fold (10^1^ to 10^2^ CFU/mL) (Table 3). The multiplex PCR assay did not detect *H. somni* DNA in spiked semen samples. In contrast, this same multiplex PCR assay detected *B. ovis*, *A. seminis*, and *H. somni* DNA simultaneously in three distinct pools of semen samples from experimentally infected rams. As expected, there was a marginal decrease in sensitivity of the multiplex PCR when compared to single PCR assays for individual agents. Thus, species-specific multiplex PCR assay detected 67% (14/21) of *B. ovis*, 87% (13/15) of *A. seminis* and 73% (11/15) of *H. somni* DNA from semen samples from experimentally infected rams that were positive by single PCR. Frequency of positivity was similar for three agents (p > 0.05). In the present study, we demonstrated that multiplex PCR assay with species specific primers does not amplify DNA from other bacteria species that can potentially cause epididymitis in rams, including *S. aureus, M. haemolytica, C. pseudotuberculosis*, and *T. pyogenes* as well as *O. anthropi*, a species phylogenetically related to *B. ovis* (Table 4). In addition, all 27 semen samples used as negative control were negative in multiplex PCR assay.

**Table 3:** **Single and multiplex PCR analytical sensitivity in semen samples spiked with 10**^**0 **^**to 10**^**6**^ **CFU/mL of *Brucella ovis*, *Atinobacillus seminis *or *Histophilus somni***

		**CFU/mL of semen**						
		**10**^**0**^	**10**^**1**^	**10**^**2**^	**10**^**3**^	**10**^**4**^	**10**^**5**^	**10**^**6**^
**Single PCR**	*B. ovis*	-	-	-	-	+	+	+
	*A. seminis*	-	+	+	+	+	+	+
	*H. somni*	-	-	-	+	+	+	+
**Multiplex PCR**	*B. ovis*	-	-	-	-	+	+	+
	*A. seminis*	-	-	+	+	+	+	+
	*H. somni*	-	-	-	-	-	-	-

+ : positive; -: negative.

**Table 4:** **Single and multiplex PCR specificity with different bacterial strains related to ovine epididymitis or phylogenetically similar to *Brucella ovis***

**Bacterial strain**	**Single PCR**			**Multiplex PCR**
	***B. ovis***	***A. seminis***	***H. somni***	
*Brucella ovis* (ATCC 25840)	+	-	-	+
*Actinobacillus seminis* (ATCC15768)	-	+	-	+
*Histophilus somni* (3384Y)	-	-	+	+
*Staphylococcus aureus* (ATCC 12600)	-	-	-	-
*Manheimia haemolitica* (D0614057)	-	-	-	-
*Corynebacterium pseudotuberculosis* (D0507204)	-	-	-	-
*Trueperella (Arcanobacterium) pyogenes* (D0602705)	-	-	-	-
*Ochrobactrum antropi* (ATCC 49188)	-	-	-	-

+ : positive; -: negative.

## Discussion

The multiplex PCR assay developed in this study was based on previously described species-specific assays for *B. ovis*[9], *A. seminis*[10], and *H. somni*[8]. Although a multiplex PCR for detection of *B. ovis*, *A. seminis*, and *H. somni* has been previously described [8] that protocol allows only for identification of *Brucella* spp. at the genus level, whereas here we describe a multiplex PCR that is the first species-specific assay.

This method proved to be a suitable diagnostic tool in cases of ovine epididymitis, which is an infectious disease that affects young and mature rams, and most of the cases are associated with *B. ovis*, *A. seminis* or *H. somni* infection. Generally, definitive diagnosis of these infections is based on bacterial isolation, which can be difficult due to lack of suitable selective media for isolation of *A. seminis* and *H. somni*[5,10]. Importantly, standardized serological tests are widely available only for the diagnosis of *B. ovis* infection [19], although serology has well documented limitations in these cases [19,20]. Furthermore, contaminating bacteria present in semen, urine and preputial wash can overgrow these pathogens, which has more fastidious growing. Therefore, PCR-based assays are considered an alternative to overcome the limitations of bacteriology [13], especially if the PCR method is direct and identifies the agent at the species level.

It is noteworthy that in the case of *B. ovis*, the multiplex PCR method developed in this study is based on amplification of sequences located in the *B. ovis* pathogenicity island 1 [21], which are absent in other *Brucella* species that infect domestic animal species [22]. Thus, the method developed in this study allows differentiation between *B. ovis* and *B. melitensis* infections as previously demonstrated [22], which is extremely relevant since these organisms are equally capable of infecting small ruminants. While *B. ovis* is considered non pathogenic for humans, *B. melitensis* has the highest zoonotic potencial among all *Brucella* species [23]. Furthermore, flocks identified as positive for *B. ovis* using the multiplex PCR method developed in this study, can be further investigated by using a more sensitive species-specific nested PCR method that has been recently developed [24].

For both experimental infections performed in this study, single PCR and bacteriology combined were used for assuring infection in experimentally challenged rams. Biological samples collected during these infections were also used to validate the multiplex PCR assay developed in this study.

In general, PCR tends to be more sensitive than bacteriology [8,9], which was also evidenced in this study, particularly in the cases of semen and blood samples. In both experimental infections performed in this study, bacteriology detected the causative agent. Although blood culture may be considered as gold standard in several bacterial infections, it may be slow and insufficiently sensitive in cases of fastidious organisms or when the bacterial load is low [25-27]. Importantly, for PCR detection only the target DNA, not viable organisms, is required for a successful diagnostic test. That accounts for part of the differences in sensitivity between the techniques used in this study, which reflects in the low agreement between these methods with some of the biological samples.

Sensitivity of the multiplex PCR tended to be slightly lower than single PCR assays for each of the three agents separately. This is expected since in individual single-targeted PCR reactions, optimal magnesium concentrations as well as optimal annealing temperatures can be applied, whereas in a multiplex reaction, magnesium concentration and annealing temperature must suit all primer pairs and target sequences, and therefore may not be quite optimal for each individual primer pair.

Semen has been used as the biological sample of choice for the diagnostic purposes in cases of ovine epididymitis [1,7-9]. As recently described for *B. ovis* infection [9], this study indicated that urine samples can be considered as alternative specimens for direct diagnosis of *A. seminis* and *H. somni* infections, although semen is the specimen of choice in the case these two agents. Blood samples were not satisfactory to detect *H. somni* infection and thus it should not be used for diagnosis of infectious ovine epididymitis.

Analytical sensitivity indicated that this species-specific multiplex PCR assay was capable of detecting DNA from *B. ovis*, *A. seminis*, and *H. somni* simultaneously, under various conditions, ensuring that the assay is also effective for diagnosis of mixed infections. The slightly lower sensitivity of this assay for detection of *H. somni* may be due to the fact that the annealing temperature employed for the multiplex PCR was optimal for the other two agents, but suboptimal for amplification of *H. somni*. Higher sensitivity of the PCR when compared to bacteriological culture has also been previously observed in rams experimentally infected with *B. ovis*[9]. Additionally, the species-specific multiplex assay was demonstrated to be specific for the target species and did not exhibit cross reactions with other organisms that may also cause infectious ovine epididymitis.

## Conclusions

The species-specific multiplex PCR assay developed in this study can be successfully used for the detection of three of the most common bacterial causes of ovine infectious epididymitis. Therefore, this technique may be a practical alternative for bacterial isolation. Moreover, urine can be used as alternative sample for DNA extraction that can be employed for the multiplex PCR method described in this study.

## Competing interests

The authors declare that they have no competing interests.

## Authors’ contributions

VM, RLS conceived and designed the study. VM, TMAS, LFC, MNX, CACJ & EAC participated in data collection and manuscript reviews. VM, TMAS are responsible of data analysis and VM for manuscript preparation. TAP participated in the supervision of data analysis and manuscript preparation and critical revision. RLS was responsible for supervision of data analysis, manuscript preparation, review, corrections and submission. All authors read and approved the final manuscript.

## Supplementary Material

Additional file 1: Table S1 Analytical sensitivity of multiplex PCR with genomic DNA from *Brucella ovis*, *Actinobacillus seminis*, and *Histophilus somni* in various concentrations and combinations. Click here for file
